# An influenza virus-triggered SUMO switch orchestrates co-opted endogenous retroviruses to stimulate host antiviral immunity

**DOI:** 10.1073/pnas.1907031116

**Published:** 2019-08-07

**Authors:** Nora Schmidt, Patricia Domingues, Filip Golebiowski, Corinna Patzina, Michael H. Tatham, Ronald T. Hay, Benjamin G. Hale

**Affiliations:** ^a^Institute of Medical Virology, University of Zurich, 8057 Zurich, Switzerland;; ^b^Centre for Gene Regulation and Expression, School of Life Sciences, University of Dundee, DD1 5EH Dundee, United Kingdom

**Keywords:** influenza, SUMO, endogenous retroviruses, dsRNA, interferon

## Abstract

Dynamic small ubiquitin-like modifier (SUMO) linkages to diverse cellular protein groups are critical to orchestrate resolution of stresses such as genome damage, hypoxia, or proteotoxicity. Defense against pathogen insult (often reliant upon host recognition of “non-self” nucleic acids) is also modulated by SUMO, but the underlying mechanisms are incompletely understood. Here, we used quantitative SILAC-based proteomics to survey pan-viral host SUMOylation responses, creating a resource of almost 600 common and unique SUMO remodeling events that are mounted during influenza A and B virus infections, as well as during viral innate immune stimulation. Subsequent mechanistic profiling focused on a common infection-induced loss of the SUMO-modified form of TRIM28/KAP1, a host transcriptional repressor. By integrating knockout and reconstitution models with system-wide transcriptomics, we provide evidence that influenza virus-triggered loss of SUMO-modified TRIM28 leads to derepression of endogenous retroviral (ERV) elements, unmasking this cellular source of “self” double-stranded (ds)RNA. Consequently, loss of SUMO-modified TRIM28 potentiates canonical cytosolic dsRNA-activated IFN-mediated defenses that rely on RIG-I, MAVS, TBK1, and JAK1. Intriguingly, although wild-type influenza A virus robustly triggers this SUMO switch in TRIM28, the induction of IFN-stimulated genes is limited unless expression of the viral dsRNA-binding protein NS1 is abrogated. This may imply a viral strategy to antagonize such a host response by sequestration of induced immunostimulatory ERV dsRNAs. Overall, our data reveal that a key nuclear mechanism that normally prevents aberrant expression of ERV elements (ERVs) has been functionally co-opted via a stress-induced SUMO switch to augment antiviral immunity.

## Significance

Primary host defenses against viruses involve specific cellular recognition of non-self nucleic acids as pathogen-associated molecular patterns (PAMPs) that trigger induction of cytokine-mediated antiviral responses. Thus, ability to discriminate between “self” and “non-self” nucleic acids, and prevent aberrant immunopathology, is a key tenet of immunity. Here, we identify self-derived endogenous retroviral RNAs as host-encoded PAMPs that are up-regulated during influenza virus infections, and which stimulate antiviral immunity. Normally, endogenous retroviruses are tightly repressed transcriptionally by host TRIM28, but infection triggers changes in the modification status of TRIM28 to alleviate repression. This provides an example of how endogenous retroviruses integrated within the host genome have been functionally co-opted by a regulatory switch to aid defense against newly invading pathogens.

A critical component of how individual cells and tissues defend themselves against invading viruses is the ability to detect the abnormality of infection and mount an appropriate antiviral response. The host innate IFN system is a powerful first line of defense against pathogens, and typically involves specific cellular recognition of non-self pathogen-associated molecular patterns (PAMPs), such as viral nucleic acids or capsids, by host pattern recognition receptors (PRRs), which then become activated to trigger expression and secretion of IFN cytokines (1). IFNs function in a paracrine or autocrine fashion to induce hundreds of IFN-stimulated gene (ISG) products that harbor diverse antiviral mechanisms and act to limit virus replication and spread (2). Key to the integrity of this cellular system is the tight discrimination of “self” from “non-self” (3), which also involves repressing, shielding, or modifying endogenous self nucleic acids with immunostimulatory potential to prevent development of aberrant immunopathologies (4–10). Nevertheless, new research is beginning to uncover previously unappreciated roles for self nucleic acid species that accumulate during the abnormal conditions of a viral infection, and which trigger activation of antiviral immunity via canonical PRRs (11–13). Such studies are driving the intriguing hypothesis that stress-induced host-derived self elements may have been co-opted to promote antiviral IFN-mediated defenses.

SUMOylation is a dynamic posttranslational modification of protein lysine residues that is crucial for regulating many cellular processes, including transcription, mRNA processing, chromatin remodeling, DNA replication, and DNA damage responses (14). Importantly, SUMOylation is highly stress-responsive, and is a critical mediator in the resolution of cellular insults, including hypoxia, heat shock, and genotoxic stresses (15). The small ubiquitin-like modifier (SUMO) system also plays key roles in the host response to viral infections (16), specifically by fine-tuning the functions of PRRs involved in the sensing of viral nucleic acids (17, 18), and by coordinating the repression of exuberant IFN stimulation (19, 20).

In this study, we sought to explore the interplay between stress-responsive SUMOylation and host antiviral defenses by conducting a proteome-wide survey of SUMO modification dynamics during infection with influenza virus variants that differ in their abilities to stimulate host IFN responses. Analysis of highly infection-regulated SUMO targets led us to focus follow-up studies on the host factor tripartite motif-containing-28 (TRIM28, also known as KAP1 or TIF1β), which has recently been implicated in contributing to inflammatory cytokine production during viral infection (21). Notably, TRIM28 is a transcriptional corepressor that acts in concert with KRAB-ZNFs (Krüppel-associated box domain-zinc finger proteins), SUMO, the histone methyltransferase SETDB1, and the nucleosome remodeling and deacetylation (NuRD) complex to induce heterochromatin formation and repress endogenous retroviral (ERV) element transcription (22–27). Such silencing of ERV elements (ERVs) is critical to limit aberrant IFN responses, as artificial derepression of ERV transcripts leads to formation of double-stranded RNA (dsRNA) that is sensed as non-self by PRRs (5–10). Here, we provide evidence that this regulatory mechanism has also been physiologically co-opted by host cells as part of a strategy to promote innate immunity: A noncanonical infection-triggered SUMO switch in TRIM28 promotes ERV expression and enhances subsequent stimulation of IFN-mediated antiviral defenses. Our data add insights into how mammals have evolved to take advantage of non-self elements integrated within their genomes, and provide an example of how traditional self versus non-self rules have been usurped in order to counteract virus infection.

## Results

### Quantitative Proteomics Identifies Conserved and Unique Features of Host SUMOylation Responses to Different Influenza Virus Infections.

**Fig. 1.**
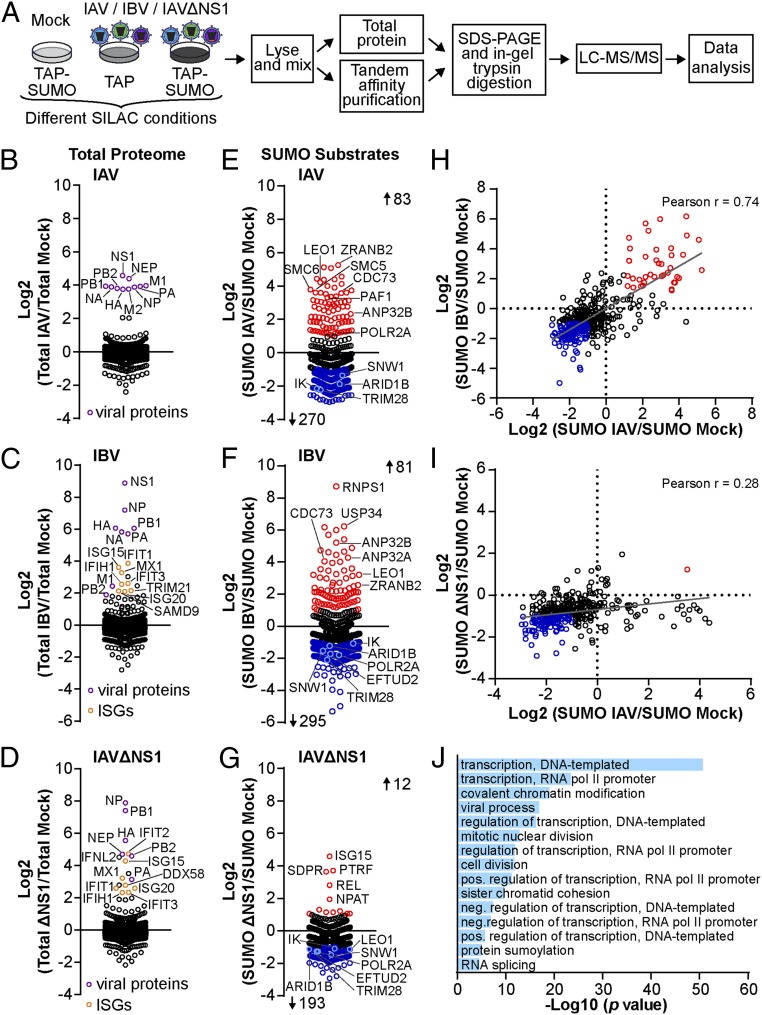
Quantitative proteomics identifies conserved and unique features of host SUMOylation responses to different influenza virus infections. (*A*) SILAC-based SUMO proteomics workflow. A549 cells expressing TAP-SUMO1, TAP-SUMO2, or TAP-tag only were grown in medium containing heavy, medium, or light isotope-labeled amino acids prior to infection, or mock, with IAV (MOI = 2 PFU per cell, 10 h) (28), IBV (MOI = 5 PFU per cell, 24 h), or IAVΔNS1 (MOI = 2 PFU per cell, 16 h). Samples of purified SUMO-modified proteins and total proteins were subjected to mass spectrometry and quantitative analysis. (*B*–*G*) Fold change in total protein abundance (*B*–*D*: purple, viral proteins; orange, ISGs with fold change ≥4) or SUMO modification (*E*–*G*: red, fold change ≥2; blue, fold change ≤−2) during infection with IAV (*B* and *E*), IBV (*C* and *F*), or IAVΔNS1 (*D* and *G*). Note: The datasets for IAV are reproduced from previous work (28). Data are averages from 2 independent experiments. (*H* and *I*) Correlation between infection-induced changes to the SUMO proteome caused by IAV and IBV (*H*) or IAV and IAVΔNS1 (*I*). (*J*) GO pathway enrichment analysis of factors that change >2-fold in SUMOylation during infection with IAV. All pathways with a *P* value < 0.001 and >5% identified pathway genes are shown. Labeling in *B*–*G* corresponds to gene names. See also Datasets S1–S3.

We extended our previous survey of the host SUMOylation response to influenza A virus (IAV) infection (28) by applying quantitative proteomic strategies to identify cellular proteins that change in SUMO modification status following infection with the distantly related influenza B virus (IBV), or an IAV strain engineered to lack expression of its major IFN-antagonist protein, NS1 (IAVΔNS1). Our previous analysis of IAV infection-induced quantitative changes to SUMO1 and SUMO2 conjugation demonstrated a very high degree of correlation between the 2 paralogs (28), indicating that data obtained from either system can represent a common and stringent consensus of SUMO substrate changes. Thus, for each virus, we conducted 2 independent “label-swap” stable isotope labeling by amino acids in cell culture (SILAC) experiments using an A549 cell line stably expressing N-terminal tandem-affinity purification (TAP)-tagged SUMO2 to determine the impact of infection on the SUMO subproteome (Fig. 1*A*). Analysis of average infection-induced changes to total proteome abundance revealed that, in contrast to IAV, both IBV and IAVΔNS1 infections triggered a classical host IFN response consisting of several ISG products, including ISG15, ISG20, MDA5, MxA, IFIT1, and IFIT3 (Fig. 1 *B*–*D* and Datasets S1–S3). This specific and rapid innate immune activation by IBV and IAVΔNS1, but not IAV, is consistent with previous reports (29), and provided us with a unique combination of experimental outcomes with which to dissect infection-induced SUMO responses. Strikingly, infection with all viruses induced widespread changes to the host SUMO proteome, which included both virus-specific increases to certain SUMO modification events as well as a common and broad loss of many SUMOylated proteins (IAV: 83 increased, 270 decreased; IBV: 81 increased, 295 decreased; IAVΔNS1: 12 increased, 193 decreased) (Fig. 1 *E*–*G* and Datasets S1–S3). Indeed, infection-induced changes to target protein SUMOylation correlated well between IAV and IBV infections, and to a limited extent between IAV and IAVΔNS1 infections, particularly with regard to the common loss of many SUMOylated proteins (Fig. 1 *H* and *I*). It is possible that constrained up-regulation of SUMOylated proteins by IAVΔNS1 (as compared to IAV) is because NS1 plays a hitherto unrecognized role in directly stimulating SUMO conjugate appearance, or because IAVΔNS1 is generally less efficient at replicating than wild-type virus. Pathway enrichment analyses of each infection-regulated SUMO proteome with DAVID (30), using all identified SUMO-modified proteins as background, revealed a remarkably similar enrichment pattern involving mainly transcriptional regulation pathways and chromatin modification (Fig. 1*J* and Datasets S1–S3). These data represent a unique and robust proteomic snapshot of host SUMO remodeling following diverse infection conditions, and will be an invaluable resource for dissecting the contribution of specific SUMOylation events to different aspects of influenza virus biology.

### Infection-Induced Loss of SUMOylated TRIM28 Is Independent of Antiviral RNA-Sensing Pathways, IFN, and Canonical DNA Damage Responses.

**Fig. 2.**
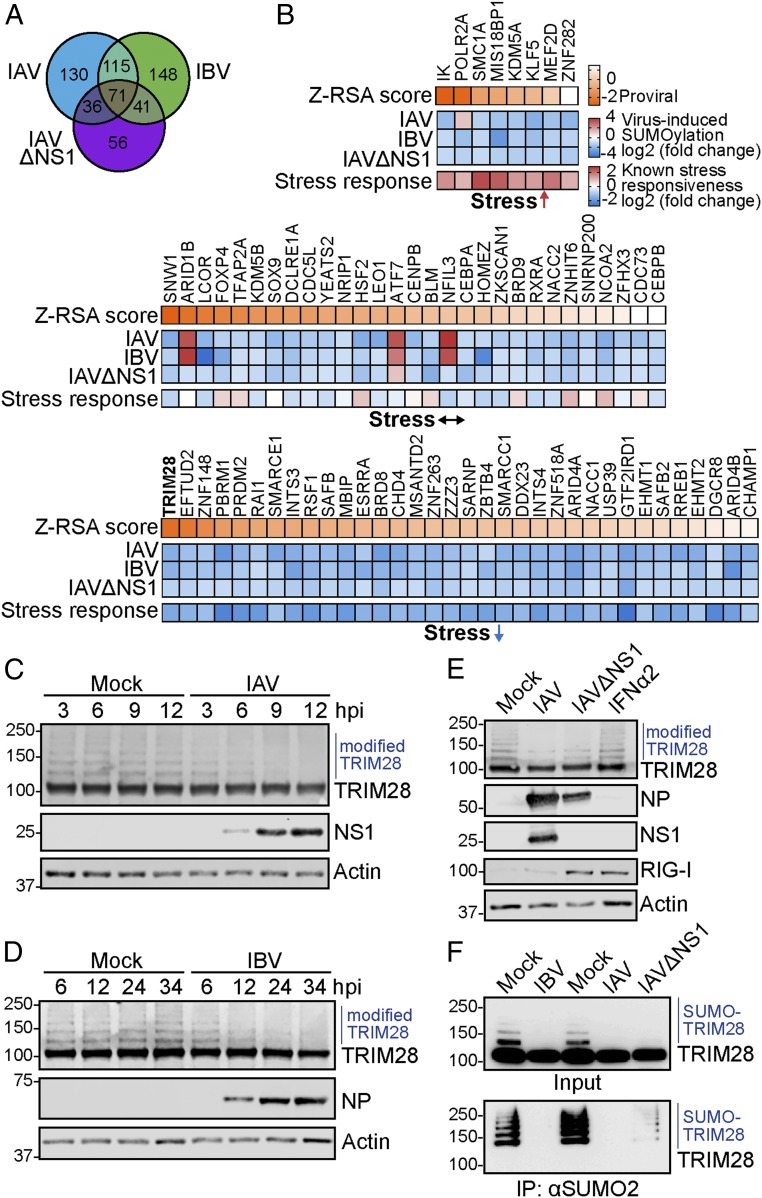
Infection-induced loss of SUMOylated TRIM28 is independent of IFN. (*A*) Venn diagram showing an overlap of factors that change in SUMOylation during infection with IAV, IBV, or IAVΔNS1. (*B*) The 71 factors that change in SUMOylation during all 3 infection conditions were grouped by their reported nonviral SUMO stress responsiveness (31) and sorted according to their known impact on IAV replication (Z-RSA score) (32). See also Dataset S4. (*C*–*E*) A549 cells infected with IAV (MOI = 5 PFU per cell) (*C*), IBV (MOI = 5 PFU per cell) (*D*), or IAV, IAVΔNS1 (MOI = 2 PFU per cell, 24 h), and IFNα2-treated (1,000 U/mL, 24 h) (*E*) were lysed, and subjected to western blot analysis for the indicated proteins. Data are representative of at least 2 independent experiments. hpi, hours postinfection. (*F*) A549 cells infected with IAV (MOI = 5 PFU per cell, 16 h), IBV (MOI = 5 PFU per cell, 24 h), or IAVΔNS1 (MOI = 5 PFU per cell, 16 h) were lysed, and SUMOylated proteins were affinity-purified using an anti-SUMO2/3 antibody. western blot was used to detect TRIM28 in input and immunoprecipitated (IP) fractions.

We focused our attention on the 71 host substrates that altered at least 2-fold in SUMO modification status during all 3 infection conditions (Fig. 2*A*). SUMOylation changes to 42 of these factors have previously been reported as responsive to nonviral cellular stresses, such as DNA damage and heat shock (31), and several are putative proviral IAV host factors based on meta-analysis of genome-wide RNAi screens (32) (Fig. 2*B* and Dataset S4). Among these was the transcriptional corepressor TRIM28. We confirmed that infection with IAV, IBV, and IAVΔNS1 leads to loss of posttranslationally modified TRIM28 (Fig. 2 *C*–*E*), and validated that this includes SUMOylated TRIM28 (Fig. 2*F*). Although TRIM28 can be phosphorylated in response to viral RNA in a PKR-dependent manner (21), infection-induced loss of posttranslationally modified TRIM28 was independent of all canonical antiviral RNA-sensing mechanisms, as IAV triggered this response even in the absence of RIG-I, MDA5, PKR, or MAVS (*SI Appendix*, Fig. S1 *A* and *B*), and IFN-α treatment alone failed to trigger this phenotype (Fig. 2*E*). Previous work has suggested that SUMOylated TRIM28 can be targeted for proteasome-mediated degradation following DNA damage-induced phosphorylation at Ser824 by the ATM kinase (33, 34). Unlike the DNA damage-inducing agent etoposide, IAV failed to trigger TRIM28 phosphorylation at Ser824, and functional inhibition of either ATM kinase (using KU55933) or the proteasome (using MG132 or lactacystin) was unable to prevent loss of posttranslationally modified TRIM28 during infection (*SI Appendix*, Fig. S1 *C*–*G*). These data suggest a previously undescribed noncanonical mechanism by which influenza virus induces loss of SUMOylated TRIM28 during infection.

### SUMOylated TRIM28 Is Required to Support Efficient Virus Replication.

**Fig. 3.**
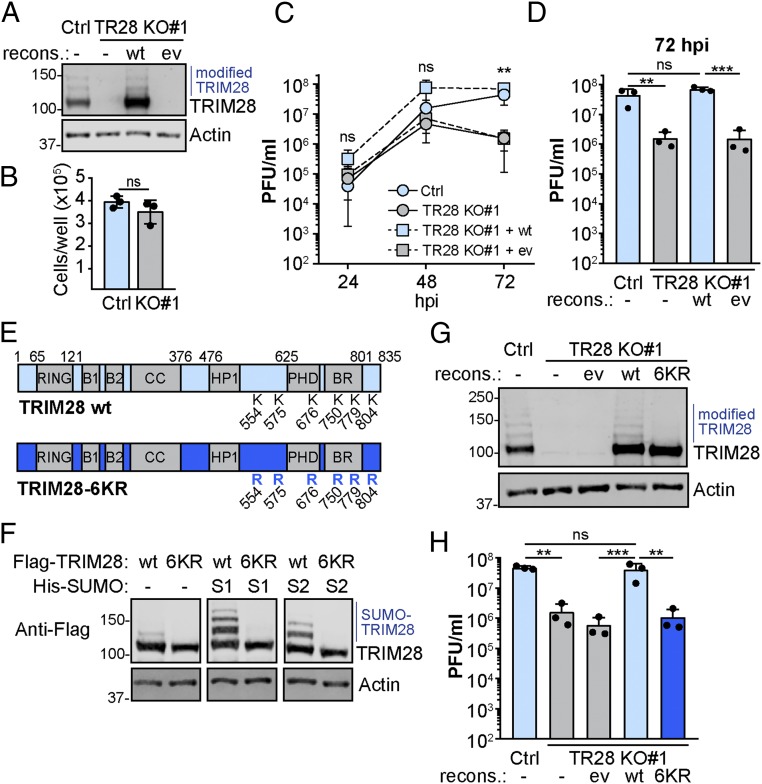
SUMOylated TRIM28 is required to support efficient virus replication. (*A*) western blot analysis of TRIM28 knockout A549 cells (TR28 KO#1), or nontargeted control A549 cells (Ctrl), reconstituted with TRIM28 or empty vector (ev) by lentiviral transduction. Data are representative of at least 2 independent experiments. (*B*) Cell number per well of the cells described in *A*, 24 h after seeding equal numbers of cells. Mean values from 3 independent experiments are plotted, with error bars representing SDs and individual data points shown. (*C* and *D*) Cells described in *A* were infected with IAV at MOI = 0.001 PFU per cell, and supernatants were collected at the indicated times prior to titration (growth curve plotted in *C*; 72 hpi only plotted in *D*). Mean values from 3 independent experiments are plotted, with error bars representing SDs. For *D*, individual data points are shown in addition. (*E*) Schematic of TRIM28 showing the RING domain, 2 B-box zinc finger domains (B1 and B2), coil-coiled domain (CC), HP1 binding site (HP1), PHD and bromo (BR) domains, and all known SUMOylation sites (K554, K575, K676, K750, K779, and K804). In the TRIM28-6KR mutant, the 6 SUMOylation sites are all changed to arginine. (*F*) Cotransfection of Flag-tagged constructs expressing wt TRIM28 or TRIM28-6KR together with His-SUMO1 or His-SUMO2 into 293T cells followed by cell lysis and western blot analysis using a Flag-specific antibody or actin antibody. Data are representative of at least 2 independent experiments. (*G*) western blot analysis of TRIM28 knockout A549 cells (TR28 KO#1), or nontargeted control A549 cells (Ctrl), reconstituted with wt TRIM28, TRIM28-6KR, or empty vector by lentiviral transduction. Data are representative of at least 2 independent experiments. (*H*) Cells described in *G* were infected with IAV at MOI = 0.001 PFU per cell, and supernatants were collected at 72 hpi prior to titration. Mean values from 3 independent experiments are plotted, error bars represent SDs, and individual data points are shown. For *B*, significance was determined by unpaired *t* test, and for *C*, *D*, and *H* by 1-way ANOVA (**P < 0.01, ***P < 0.001; ns, nonsignificant).

To analyze the role of TRIM28 during influenza virus replication, we established A549-based TRIM28 knockout (KO) cell lines using CRISPR/Cas9 (Fig. 3*A* and *SI Appendix*, Fig. S2*A*). There was no apparent cell-growth defect in TRIM28-KO cells as compared with control (Ctrl) cells (Fig. 3*B*). However, multicycle viral growth analysis revealed inefficient replication of IAV in 2 independent TRIM28-KO clones as compared with Ctrl, particularly at late times postinfection (Fig. 3 *C* and *D* and *SI Appendix*, Fig. S2 *B* and *C*). This phenotype could be reversed by reconstituting the TRIM28-KO clones with wild-type (wt) TRIM28 via lentiviral transduction (Fig. 3 *A*, *C*, and *D* and *SI Appendix*, Fig. S2), indicating a specific effect. We used this knockout and reconstitution system to understand the impact that infection-triggered loss of SUMOylated TRIM28 might have on IAV replication. Six lysine residues in TRIM28 have previously been reported to be major targets for SUMO modification (26, 27). We created a TRIM28 mutant construct in which all these 6 lysines were substituted to arginine (6KR; Fig. 3*E*) and validated that this TRIM28-6KR construct could no longer be modified by SUMO1/2 (Fig. 3*F*). All 6 lysines had to be simultaneously substituted to arginine to fully ablate TRIM28 SUMOylation, as TRIM28 constructs retaining various combinations of lysines maintained specific SUMOylation profiles (*SI Appendix*, Fig. S3 *A* and *B*). Strikingly, reconstitution of TRIM28-KO cells with the TRIM28-6KR construct failed to result in efficient IAV replication as compared with reconstitution with wt TRIM28 (Fig. 3 *G* and *H*). Reconstitution of TRIM28-KO cells with other TRIM28 mutant constructs that either retained some SUMOylation or were lacking the N-terminal RING or C-terminal bromo domains resulted in partial rescue phenotypes (*SI Appendix*, Fig. S3), implying minor contributory roles for these domains. Notably, reconstitution of TRIM28-KO cells with phosphomimetic or phospho-ablation mutants of TRIM28 at serine 473 or 824 conferred IAV replication efficiencies indistinguishable from wt TRIM28 (*SI Appendix*, Fig. S4), and IAV-triggered loss of SUMOylated TRIM28 was not affected by S824A or S824D substitutions (*SI Appendix*, Fig. S4*D*), consistent with our inhibitor studies. These data indicate that complete TRIM28 SUMOylation, but not phosphorylation, supports efficient viral growth, and suggested to us that noncanonical infection-triggered loss of SUMOylated TRIM28 may be a cellular response to limit virus replication.

### Cells Expressing SUMOylation-Deficient TRIM28 Exhibit Up-Regulation of ZNF Family Genes and an Enhanced Innate Immune Defense Gene Signature.

**Fig. 4.**
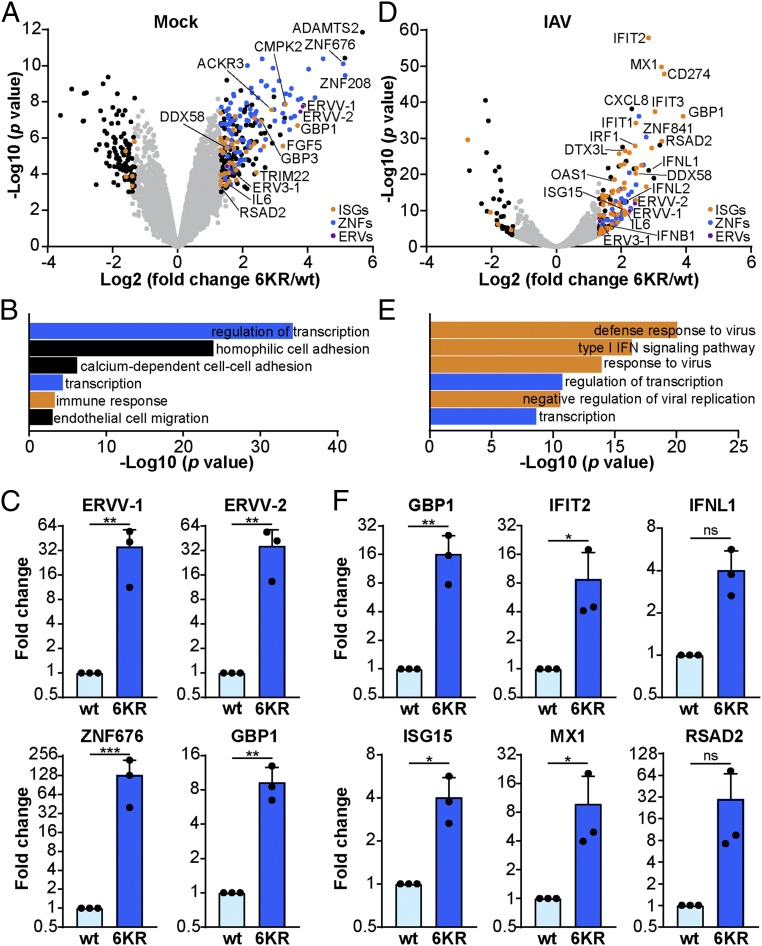
Cells expressing SUMOylation-deficient TRIM28 exhibit an increased innate immune defense gene signature and are primed for triggering enhanced IFN-stimulated responses during infection. (*A* and *D*) Transcriptome analysis comparing TRIM28-KO A549 cells reconstituted with wt TRIM28 or TRIM28-6KR, which were mock infected (*A*) or infected with IAV (MOI = 10 PFU per cell, 6 h) (*D*). Data are derived from 3 independent replicates. Genes with a *P* < 0.001 and fold change 6KR/wt >2.5 or <−2.5 are depicted in blue (zinc finger proteins), orange (IFN-stimulated genes), purple (ERVs), or black (others). ISGs were identified using an online database [http://isg.data.cvr.ac.uk (62)]. (*B* and *E*) GO pathway enrichment analysis of genes significantly up-regulated (*P* < 0.001 and fold change > 2.5) in cells expressing TRIM28-6KR under mock (*B*) or infected (*E*) conditions, showing the 6 most significantly enriched pathways. (*C* and *F*) Differential expression of selected genes was validated by RT-qPCR analysis under mock (*C*) or IAV infection (*F*) conditions as described in *A* and *D*. Bars represent mean values and SDs of 3 independent experiments (each dot represents 1 replicate). Significance was determined by unpaired *t* test (*P < 0.05, **P < 0.01, ***P < 0.001; ns, nonsignificant). See also Datasets S5 and S6.

TRIM28 SUMOylation has previously been implicated in regulating its transcriptional corepressor activity (26, 27). We therefore performed transcriptomic analyses on TRIM28-KO cells reconstituted with either wt TRIM28 or TRIM28-6KR to identify genes differentially expressed between the 2 conditions that could be candidates regulated by TRIM28 SUMOylation. A large number of ZNF-related genes (associated with transcriptional regulation), as well as 3 different ERV envelope genes (*ERVV-1*, *ERVV-2*, and *ERV3-1*), were more highly expressed in the TRIM28-6KR cells as compared with wt TRIM28 cells (Fig. 4 *A* and *B* and Dataset S5). This is consistent with recent findings identifying such genes as repression targets for TRIM28 (9). In addition, we also detected higher expression of a small number of innate immunity-related genes, exemplified by ISGs, in the TRIM28-6KR cells as compared with wt TRIM28 cells. These included *GBP1*, *TRIM22*, *RSAD2* (viperin), and *DDX58* (RIG-I) (Fig. 4 *A* and *B* and Dataset S5). These transcriptomic results were independently confirmed using RT-qPCR assays specific for several differentially expressed genes (Fig. 4*C*).

### Cells Expressing SUMOylation-Deficient TRIM28 Are Primed for Triggering Enhanced IFN-Stimulated Responses during Infection.

Given the surprising result that cells expressing SUMOylation-deficient TRIM28 exhibited an enhanced innate immune gene signature, including increased expression of *DDX58* (RIG-I), a known antiviral sensor for influenza viruses (35), we next performed transcriptomic analysis of IAV-infected wt TRIM28 and TRIM28-6KR cells. Strikingly, IAV-infected TRIM28-6KR cells were characterized by a much greater induction of ISGs than IAV-infected wt TRIM28 cells (Fig. 4*D* and Dataset S6). This result was also reflected by looking at significantly enriched pathways in IAV-infected TRIM28-6KR cells as compared with IAV-infected wt TRIM28 cells, which included “defense response to virus” and “type I IFN signaling” (Fig. 4*E* and Dataset S6). Differential gene expression was confirmed for several factors by specific RT-qPCR (Fig. 4*F*) and western blot following IAVΔNS1 infection (*SI Appendix*, Fig. S5). Overall, these data suggested that SUMOylation-deficient TRIM28 is unable to assist in the repression of some canonical innate immune response genes, the enhanced expression of which may act to prime cells for increased antiviral gene expression following infection.

### Cells Expressing SUMOylation-Deficient TRIM28 Display Constitutive Derepression of ERV Elements.

**Fig. 5.**
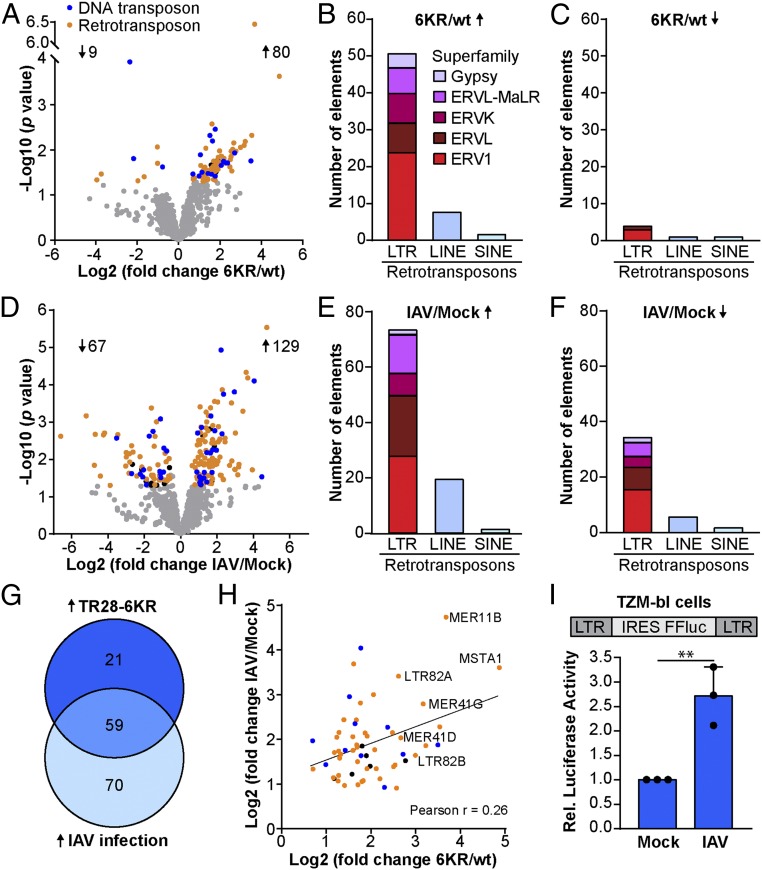
Up-regulation of transposable elements in cells expressing SUMOylation-deficient TRIM28 and during IAV infection. (*A* and *D*) Transcriptome datasets from Fig. 4 were reanalyzed for expression of repetitive elements comparing TRIM28-KO A549 cells reconstituted with wt TRIM28 versus TRIM28-6KR (*A*), or mock versus IAV-infected TRIM28-wt A549 cells (*D*). Elements with a *P* value < 0.05 and fold change >1.5 or <−1.5 are depicted in blue (DNA transposons), orange (retrotransposons), or black (others). (*B*, *C*, *E*, and *F*) Subclasses of retrotransposons that were significantly up- or down-regulated in TRIM28-6KR–expressing cells (*B* and *C*) or during IAV infection (*E* and *F*): LINE, long interspersed nuclear element; LTR, long-terminal repeat retrotransposon; SINE, short interspersed nuclear element. (*G*) Overlap analysis of TEs that are significantly up-regulated in TRIM28-6KR–expressing cells (TR28-6KR) and during IAV infection. (*H*) Fold-change correlation between the 59 TEs that are up-regulated in TRIM28-6KR cells and during IAV infection. Elements are depicted in blue (DNA transposons), orange (retrotransposons), or black (others). (*I*) TZM-bl cells that express *Firefly* luciferase under control of an LTR were infected with IAV (MOI = 50 PFU per cell) or mock infected. *Firefly* luciferase was measured at 16 hpi. Bars represent mean values and SDs of 3 independent experiments (each dot represents 1 replicate). Significance was determined by unpaired *t* test (**P < 0.01). See also Dataset S7.

Recent depletion studies of TRIM28 and its associated proteins, such as SETDB1, have revealed derepression of ERV elements in the absence of these factors, and an associated triggering of innate immune responses (6, 9). Mechanistically, this is due to bidirectional transcription of ERVs and the subsequent formation of immunostimulatory dsRNAs from annealing of the resulting transcripts (6, 10, 36). Given the parallels of these previous observations with our own, together with the noted increase in expression of 3 ERV genes in our TRIM28-6KR transcriptome dataset, we sought to investigate whether lack of SUMOylated TRIM28 leads to transcriptional activation of ERVs that could have immunostimulatory activity. To this end, we reanalyzed our transcriptome datasets using a method which allows mapping of repetitive sequences to the genome and thereby identification of transposable elements (TEs), such as ERVs, usually excluded from typical RNA-seq experiments (37). Notably, as our initial transcriptome data were acquired following the standard procedure of polyA enrichment, only polyadenylated TEs can be identified in this way. Nevertheless, using this method, we found a large number of DNA transposons and retrotransposons significantly up-regulated in TRIM28-KO cells reconstituted with TRIM28-6KR as compared with reconstituted with wt TRIM28 (Fig. 5*A* and Dataset S7). Among the up-regulated retrotransposons, most appeared to be long-terminal repeat (LTR) elements that belong to the ERV1, ERVL, and ERVK subfamilies (Fig. 5 *B* and *C*). These data indicate that SUMOylated TRIM28, unlike SUMOylation-deficient TRIM28, is normally able to repress expression of TEs, including ERVs.

### Derepression of ERV Elements Occurs during IAV Infection.

In our phenotypic studies, we had used the SUMOylation-deficient TRIM28 construct (TRIM28-6KR) as a surrogate to mimic the effects of virus-triggered loss of SUMOylated TRIM28 on host cell biology. We therefore reasoned that IAV infection, as it leads to loss of SUMOylated TRIM28, should have similar biological consequences and lead to up-regulation of ERVs. Indeed, previous studies have found up-regulation of ERV elements during both IAV and other virus infections (38, 39). We reanalyzed our IAV-infected transcriptome datasets to allow mapping of repetitive sequences, and found that IAV infection (similar to expression of SUMOylation-deficient TRIM28) led to up-regulation of many cellular TEs (Fig. 5 *D* and *E* and Dataset S7), although it is notable that some TEs from the same subfamilies are also suppressed during infection (Fig. 5*F*). Overlap analysis revealed that 59 TEs were up-regulated by both IAV infection and expression of the SUMOylation-deficient TRIM28 construct (Fig. 5*G*), and the degree of expression between these 2 independent conditions showed a correlative trend (Fig. 5*H*). We also confirmed that SUMO-modified TRIM28 is lost during IAV infection of primary-like diploid MRC-5 cells concomitant with *ERVV-1* and *ERVV-2* induction, suggesting widespread up-regulation of cellular TEs in primary human cells during infection, similar to that observed in A549 cells (*SI Appendix*, Fig. S6). TRIM28 was recently shown to be a repressor of HIV-1 (40, 41), and knockdown of TRIM28 increases expression of HIV-LTR–driven *Firefly* luciferase in TZM-bl cells (41). In agreement with our bioinformatic analyses, IAV infection [similar to TRIM28 depletion (41)] also induced expression of HIV-LTR–driven *Firefly* luciferase in TZM-bl cells (Fig. 5*I*). These combined analyses suggest that IAV-triggered loss of SUMOylated TRIM28 causes derepression of ERVs during infection.

### Antiviral Activity of SUMOylation-Deficient TRIM28 Relies on IFN Induction and Signaling.

**Fig. 6.**
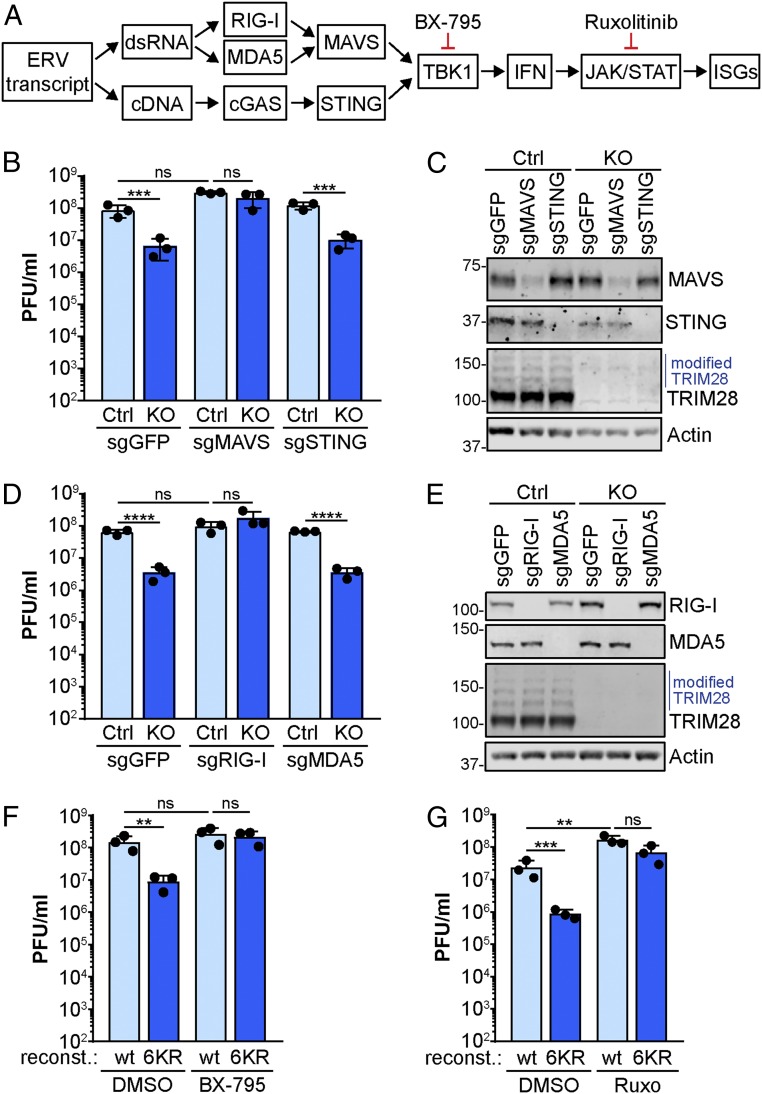
Antiviral activity of SUMOylation-deficient TRIM28 relies on dsRNA-mediated IFN induction and signaling pathways. (*A*) Schematic showing the canonical IFN signaling cascade triggered by ERV transcripts and leading to ISG expression. Stages of action of specific small-molecule inhibitors are shown. (*B*) TRIM28-KO (and Ctrl) A549 cells were transduced with lentiviruses expressing Cas9 and 3 different sgRNAs targeting MAVS, STING, or GFP (negative control). Selected cell pools were subsequently infected with IAV (MOI = 0.001 PFU per cell) and supernatants were collected at 72 hpi prior to titration. (*C*) Cells generated for *B* were processed for western blot to assess knockout efficiency. Data are representative of at least 2 independent experiments. (*D*) TRIM28-KO (and Ctrl) A549 cells were transduced with lentiviruses expressing Cas9 and 3 different sgRNAs targeting RIG-I, MDA5, or GFP (negative control). Selected cell pools were subsequently infected with IAV (MOI = 0.001 PFU per cell) and supernatants were collected at 72 hpi prior to titration. (*E*) Cells generated for *D* were treated with IFNα2 (1,000 U/mL, 16 h) and processed for western blot to assess knockout efficiency. Data are representative of at least 2 independent experiments. (*F* and *G*) TRIM28-KO A549 cells reconstituted with wt TRIM28 or TRIM28-6KR were infected with IAV (MOI = 0.001 PFU per cell) in the presence or absence of the TBK1 inhibitor BX-795 (0.5 µM; *F*) or the JAK1 inhibitor ruxolitinib (4 µM; *G*) for 72 h, prior to titration of supernatants. For *B*, *D*, *F*, and *G*, mean values from 3 independent experiments are plotted, error bars represent SDs, and individual data points are shown. Significance was determined by 1-way ANOVA (**P < 0.01, ***P < 0.001, ****P < 0.0001; ns, nonsignificant).

Given that SUMOylation-deficient TRIM28 failed to suppress expression of ERVs, as well as expression of an innate immune defense gene signature, we sought to determine if, and how, these activities were responsible for mediating the antiviral function of SUMOylation-deficient TRIM28 against IAV. ERV transcription is usually thought to stimulate MAVS-based innate immune responses as dsRNA (via bidirectional transcription and annealing of cRNA transcripts), but cGAS-STING agonistic cDNA can also be generated by reverse-transcriptase activity (42) (Fig. 6*A*). To dissect these 2 possibilities in our system, we applied CRISPR/Cas9 to our existing TRIM28-KO (and Ctrl) cells to generate MAVS or STING KOs in the presence or absence of TRIM28. Strikingly, knockout of MAVS, but not STING, fully restored the replication of IAV in TRIM28-KO cells, suggesting that TRIM28-repressed ERVs potentiate a MAVS-dependent dsRNA-mediated response (Fig. 6 *B* and *C*). A similar strategy revealed that this effect was also dependent upon RIG-I, but not MDA5 (Fig. 6 *D* and *E*). To expand these observations to the functions of SUMOylation-deficient TRIM28, we made use of commercially available small-molecule inhibitors of the dsRNA-mediated IFN induction cascade (BX-795, an inhibitor of TBK1) and the IFN signaling cascade (ruxolitinib, an inhibitor of JAK1) (Fig. 6*A*). Notably, treatment with either inhibitor restored the ability of IAV to replicate efficiently in TRIM28-KO cells reconstituted with TRIM28-6KR, so that replication was similar to TRIM28-KO cells reconstituted with wt TRIM28 (Fig. 6 *F* and *G*). These data indicate that SUMOylation-deficient TRIM28, which cannot restrain aberrant immunostimulatory ERV expression, likely stimulates and primes IFN-mediated innate immune defenses to restrict IAV replication via a RIG-I–, MAVS-, TBK1-, and JAK1-dependent pathway.

## Discussion

Herein, we aimed to provide a comprehensive proteomic survey of host SUMOylation responses to diverse influenza virus infection states, including under conditions where virus-mediated innate immune suppression has been abrogated. This resource, encompassing dynamic SUMO modification changes to almost 600 host proteins, will permit future investigations into the contribution of specific SUMOylation events to both influenza virus biology and innate immunity.

**Fig. 7.**
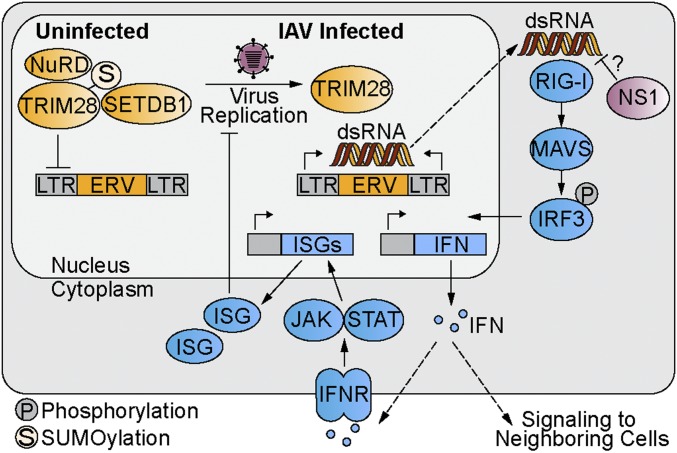
Model of the infection-triggered TRIM28 SUMO switch leading to increased antiviral responses. Upon loss of SUMOylated TRIM28, transcriptional repression of ERVs is released, leading to formation of dsRNA that is sensed by cellular PRRs, such as RIG-I, which induce activation of the canonical antiviral IFN system. Viral dsRNA-binding proteins, such as NS1, may act as antagonists of this pathway.

The major experimental focus of this study was to determine the consequences of infection-triggered loss of SUMOylated TRIM28, a cellular reaction to all 3 influenza virus infection states that we found to be independent of canonical innate immune stimulation and DNA damage-like stimuli involving the ATM kinase. Thus, the infection-triggered loss of SUMOylated TRIM28 observed here does not appear to be related to well-characterized phosphorylation-dependent switches in TRIM28 activities, which have previously been linked to regulating DNA virus latency, DNA damage repair, the cell cycle, and inflammatory responses to infection (21, 43, 44). Indeed, TRIM28 SUMOylation, but not phosphorylation, has been specifically implicated in repression of transcription from integrated retroviral genomes (45), indicating phenotypically distinct activities for different TRIM28 posttranslational modifications. By combining genetics, transcriptomics, and classical virology, we report that infection-triggered loss of SUMOylated TRIM28 constitutes a previously unrecognized mechanism for the cell to mount innate immune defenses. Our data suggest a model (Fig. 7) whereby loss of SUMOylated TRIM28 disrupts its ability to form a functional repressor complex, causing the predominant transcriptional up-regulation of LTR-containing ERV elements. ERVs have been reported to exhibit bidirectional transcription, with the subsequent transcripts annealing to form immunostimulatory dsRNAs (6, 10, 36). In line with this, we provide evidence that engineered deficiency in SUMOylated TRIM28 leads to an enhanced IFN-mediated antiviral response acting via canonical components of dsRNA-sensing pathways, such as RIG-I, MAVS, TBK1, and JAK/STAT. These observations support a model whereby this infection-triggered SUMO switch leads to aberrant up-regulation of self ERV RNAs that are sensed as non-self by PRRs (Fig. 7). Notably, while RIG-I (but not MDA5) is the critical sensor of influenza virus genomes (35), MDA5 can play a clear antiviral role against influenza viruses (46), and other studies have implicated ERV-derived dsRNA as both an MDA5 and RIG-I agonist (5–7). We therefore do not rule out that both RIG-I and MDA5 may act to sense IAV infection-triggered increases in self ERV-derived dsRNA to promote antiviral activity. A further tempting speculation to make is that infection–up-regulated ERV dsRNA is a target for the major influenza virus IFN-antagonist protein NS1, whose viral and host dsRNA targets have remained enigmatic. Indeed, it is clear from the proteomic work presented here that wild-type IAVs generally do not induce an innate immune response, despite triggering up-regulation of immunostimulatory ERV RNAs. Thus, wild-type IAVs must be able to mitigate the antiviral effects of ERV up-regulation, and we hypothesize that the sequence-independent dsRNA-binding activity of IAV NS1, which can oligomerize around both long and short dsRNAs (47), antagonizes IFN induction in response to ERV RNA (Fig. 7). Alternatively, NS1 (and other viral factors) might directly inhibit ERV-mediated activation of RIG-I/MAVS signaling (48).

The viral component that triggers the cellular SUMO switch stress response in TRIM28 has yet to be precisely defined, but viral polymerase activity could play a role given that this nuclear event can cause widespread SUMO remodeling (28). Future work to determine the specific viral trigger for this reaction, as well as the cellular factors driving the loss of SUMOylated TRIM28, will have important implications for our understanding of the molecular players that fully contribute to host protection against viruses.

In sum, the infection-triggered SUMO switch mechanism described here provides a new example of how the traditional clear-cut paradigm of cells detecting self versus non-self is being eroded, and highlights the important emerging roles that stress-induced self RNAs play in innate immune defenses (11–13). Our data contribute to the increasing recognition that ERV sequences have been co-opted as critical orchestrators of host antiviral immunity (49, 50).

## Materials and Methods

### Cells and Viruses.

A549, MDCK, 293T, and TZM-bl cells (51) were cultured in Dulbecco’s modified Eagle’s medium (DMEM) (Life Technologies) supplemented with 10% (vol/vol) FCS, 100 units/mL penicillin, and 100 μg/mL streptomycin (Gibco Life Technologies). MRC-5 cells were cultured in minimum essential medium Eagle supplemented with 10% (vol/vol) FCS, 100 units/mL penicillin, 100 μg/mL streptomycin, 2 mM l-glutamine, and 1% (vol/vol) nonessential amino acids (Life Technologies). A549 cells stably expressing TAP-tagged SUMO1 or SUMO2 were described previously (28). To generate TRIM28-KO and control cells, A549 cells were transiently transfected with pSpCas9.sgTRIM28-2A-GFP or pSpCas9.sgFfluc-2A-GFP plasmids (see below) using Lipofectamine 3000 (ThermoFisher) at a 1:1 DNA:transfection reagent ratio. GFP-positive cells were obtained by FACS and seeded at 100 cells per 10-cm dish. Clonal cell colonies were expanded and screened for TRIM28 expression by western blot. To reintroduce expression of wt TRIM28 or mutants, cells were transduced with lentiviral particles produced by cotransfection of 293T cells with the respective pLVX-IRES-Puro (Clontech)–derived construct, pMD2.G, and pCMVdR8.91. Forty-eight hours after transduction, cells were subjected to puromycin selection (1 µg/mL). Influenza virus stocks A/WSN/33 (IAV), A/WSN/33 ΔNS1 (IAVΔNS1), and B/Yamagata/88 (IBV) were grown in MDCKs or eggs (as appropriate).

### Plasmids.

To generate constructs for the CRISPR/Cas9 systems, oligos encoding single-guide RNAs (sgRNAs) (Dataset S8) were annealed, phosphorylated, and inserted into lentiCRISPRv2 plasmid [gift from Feng Zhang, Massachusetts Institute of Technology, Boston MA; Addgene plasmid 52961 (52)] or pSpCas9(BB)-2A-GFP [gift from Feng Zhang; Addgene plasmid 48138 (53)]. To generate TRIM28 expression plasmids, TRIM28 was PCR-amplified from pEGFP-TRIM28 [gift from Fanxiu Zhu, Florida State University, Tallahassee, FL; Addgene plasmid 45568 (54)] and inserted between the EcoRI and XbaI sites of p3xFLAG-CMV-7.1 (Sigma-Aldrich). TRIM28-6KR, TRIM28 N-3KR, TRIM28 3KR-C, and TRIM28 delBR mutants were generated by subsequent site-directed mutagenesis (QuikChange II XL; Agilent Technologies). The TRIM28 delRING mutant was generated by PCR, where nucleotides 1 to 192 and nucleotides 364 to 2,508 were amplified in the first PCR, followed by a second PCR to fuse the 2 fragments together. All primer sequences are listed in Dataset S8. As necessary, inserts were subcloned into pLVX-IRES-Puro. The identity of inserts in each construct was confirmed by Sanger sequencing. pLVX-6His-SUMO1 and pCAGGS-6His-SUMO2 plasmids were described previously (55).

### Transient Transfection.

Generally, 1.5 × 10^5^ 293T cells were seeded per well of a 24-well plate and transfected the next day with 300 to 500 ng of each of the indicated constructs using FuGENE HD transfection reagent (Promega) at a 1:3 DNA:transfection reagent ratio.

### Lenti-CRISPR Knockout System.

293T cells were transfected with a pool of 3 lentiCRISPRv2 plasmids targeting each gene of interest (Dataset S8), together with the appropriate packaging plasmids. At 48 h posttransfection, the supernatant was harvested and used to transduce A549 cells prior to puromycin selection (1 µg/mL).

### Virus Infection.

Generally, 2 × 10^5^ cells were seeded per well of a 12-well plate and infected the next day at the indicated multiplicity of infection (MOI). Cells from separate wells were trypsinized and counted to determine cell number. Virus inoculum was prepared in PBS supplemented with 0.3% BSA, 1 mM Ca^2+^/Mg^2+^, 100 units/mL penicillin, and 100 μg/mL streptomycin. Cells were incubated with the inoculum for 1 h, washed 3 times with PBS, and then overlaid with DMEM supplemented with 0.1% FBS, 0.3% BSA, 20 mM Hepes, 100 units/mL penicillin, and 100 μg/mL streptomycin. Where indicated, IFNα2a (Roferon-A; Roche), etoposide (Sigma-Aldrich; E1383), KU55933 (Sigma-Aldrich; SML1109), MG132 (Sigma-Aldrich; M7449), lactacystin (Enzo Life Sciences; BML-PI104-0200), ruxolitinib (Santa Cruz; sc-364729), or BX-795 (Sigma-Aldrich; SML0694) was added to the medium at the indicated concentration. Virus titers in the supernatants at the indicated time points were determined by standard plaque assay on MDCK cells. All infections with IBV were performed at 33 °C, while 37 °C was used for all other conditions.

### SILAC, TAP, Mass Spectrometry, and Bioinformatic Analysis.

Mass spectrometry analyses of SUMOylated proteins following the indicated infections were performed as described previously (28). Infection times differed for each virus to allow maximal replication before appearance of any cytopathic effects. Data processing was carried out according to previously described protocols (28), with all cutoffs and processing steps detailed on the “Info” tabs of each appropriate table (Datasets S1–S3). The raw mass spectrometry proteomics data have been deposited in the ProteomeXchange Consortium via the PRIDE (56) partner repository with the dataset identifier PXD014136. Pathway enrichment analyses were performed using DAVID (30).

### Western Blot Analysis.

Cell lysates were generated and processed according to previously described protocols (28). Proteins were transferred to nitrocellulose membranes (Amersham) and detected using the following primary antibodies: β-actin (Santa Cruz; sc-47778), TRIM28 (Bethyl; A300-27A), phospho-TRIM28 Ser824 (abcam; ab70369), IAV-NS1 (pAb 155; gift from Peter Palese, Icahn School of Medicine at Mount Sinai, New York, NY), IBV-NP (abcam; ab20711), RIG-I (gift from Adolfo García-Sastre, Icahn School of Medicine at Mount Sinai, New York, NY), PKR (abcam; ab32052), MDA5 (Cell Signaling; 5321), MAVS (Cell Signaling; 2992), STING (Cell Signaling; 13647), FLAG M2 (Sigma; F1804), and ubiquitin FK2 (Millipore; 04-263).

### SUMO Immunoprecipitation Assay.

SUMOylated proteins were enriched by immunoprecipitation as described previously (28).

### Transcriptome Analysis by Illumina RNA Sequencing.

#### Library preparation.

Total RNA from 3 independent replicates was isolated and quality was determined using a Qubit 1.0 fluorometer (Life Technologies) and Bioanalyzer 2100 (Agilent). TruSeq RNA Sample Prep Kit v2 (Illumina) was used in subsequent steps. Briefly, total RNA (100 to 1,000 ng) was polyA-enriched before reverse transcription into double-stranded cDNA. cDNA samples were fragmented, end-repaired, and polyadenylated before ligation of TruSeq adapters containing the multiplexing index. Fragments containing TruSeq adapters on both ends were selectively enriched with PCR. Quality and quantity of the enriched libraries were validated using a Qubit 1.0 fluorometer and LabChip GX (Caliper Life Sciences). The product was a smear with an average fragment size of ∼260 bp. Libraries were normalized to 10 nM in 10 mM Tris⋅Cl (pH 8.5) with 0.1% Tween 20. Adapter sequences for TruSeq RNA and DNA Sample Prep Kits are listed in Dataset S8.

#### Cluster generation and sequencing.

The TruSeq PE Cluster Kit HS4000 or TruSeq SR Cluster Kit HS4000 (Illumina) was used for cluster generation using 10 pM pooled normalized libraries on the cBOT. Sequencing was performed on the Illumina HiSeq 4000 (single-end, 125 bp) using the TruSeq SBS Kit HS4000 (Illumina).

#### Data analysis.

Reads were quality-checked with FastQC. Sequencing adapters were removed with Trimmomatic (57) and reads were subjected to hard trimming by 5 bases at the 3′ end. Successively, reads >20 bases long, and with an overall average phred quality score >10, were aligned to the reference genome and transcriptome of *Homo sapiens* (FASTA and GTF files, respectively, downloaded from GRCh38) with STAR v2.5.1 (58), with default settings for single-end reads. Distribution of the reads was quantified using the R package GenomicRanges (59), and differentially expressed genes were identified using the R package edgeR (60) (both from Bioconductor version 3.0). The RNA-seq data have been deposited in NCBI’s Gene Expression Omnibus (61) and are accessible through GEO series accession no. GSE133329.

#### Repetitive element analysis.

Analysis of repetitive elements was performed as described previously (37).

### RT-qPCR Analysis.

Cellular RNA was extracted using the ReliaPrep RNA Cell Kit (Promega) and cDNA was synthesized from 1 µg RNA using SuperScript III reverse transcriptase (ThermoFisher) and random primers (Promega). cDNA was diluted 1:5 with water and RT-qPCR was performed using Fast EvaGreen qPCR Master Mix (Biotium) and 0.2 µM forward and reverse primers (listed in Dataset S8) in the 7300 Real-Time PCR System (Applied Biosystems) with the following thermal cycling protocol: 10 min at 95 °C, 40 cycles of 15 s at 95 °C and 1 min at 60 °C, followed by a dissociation stage from 60 to 95 °C to confirm primer specificity. The relative gene expression was calculated with the ΔΔC_t_ method, using either GAPDH or 18S rRNA for normalization.

### Luciferase Assay.

TZM-bl cells (4 × 10^4^ per well) were seeded in a 96-well plate and infected with IAV (or mock) as indicated. At 16 h postinfection, *Firefly* luciferase activity was analyzed using the ONE-Glo Luciferase Assay System (Promega) according to the manufacturer’s instructions.

### Statistical Analysis.

Statistical analyses were performed using GraphPad Prism 7 software. Virus titer data were log-transformed and analyzed by unpaired 2-tailed *t* test or 1-way ANOVA for comparison of multiple conditions. For RT-qPCR data, ΔCt values were analyzed by unpaired 2-tailed *t* test. The *P* values for significance are stated in the figure legends.

## Supplementary Material

Supplementary File

Supplementary File

Supplementary File

Supplementary File

Supplementary File

Supplementary File

Supplementary File

Supplementary File

Supplementary File
