# Supplementary material for: An influenza virus-triggered SUMO switch orchestrates co-opted endogenous retroviruses to stimulate host antiviral immunity
Source: Proc Natl Acad Sci U S A. 2019 Aug 7;116(35):17399–408. doi: 10.1073/pnas.1907031116 (PMC6717285; doi:10.1073/pnas.1907031116)
Supplement: Supplementary File [file pnas.1907031116.sapp.pdf]

PNAS
www.pnas.org

Supplementary Information for

An Influenza Virus Triggered SUMO Switch Orchestrates Co-Opted Endogenous Retroviruses to Stimulate Host Antiviral Immunity

Nora Schmidt, Patricia Domingues, Filip Golebiowski, Corinna Patzina, Michael H. Tatham, Ronald T. Hay & Benjamin G. Hale

Corresponding author: Benjamin G. Hale
Email: hale.ben@virology.uzh.ch

This PDF file includes:

Figs. S1 to S6

Other supplementary materials for this manuscript include the following:

Additional Datasets S1 to S8

www.pnas.org/cgi/doi/10.1073/pnas.1907031116

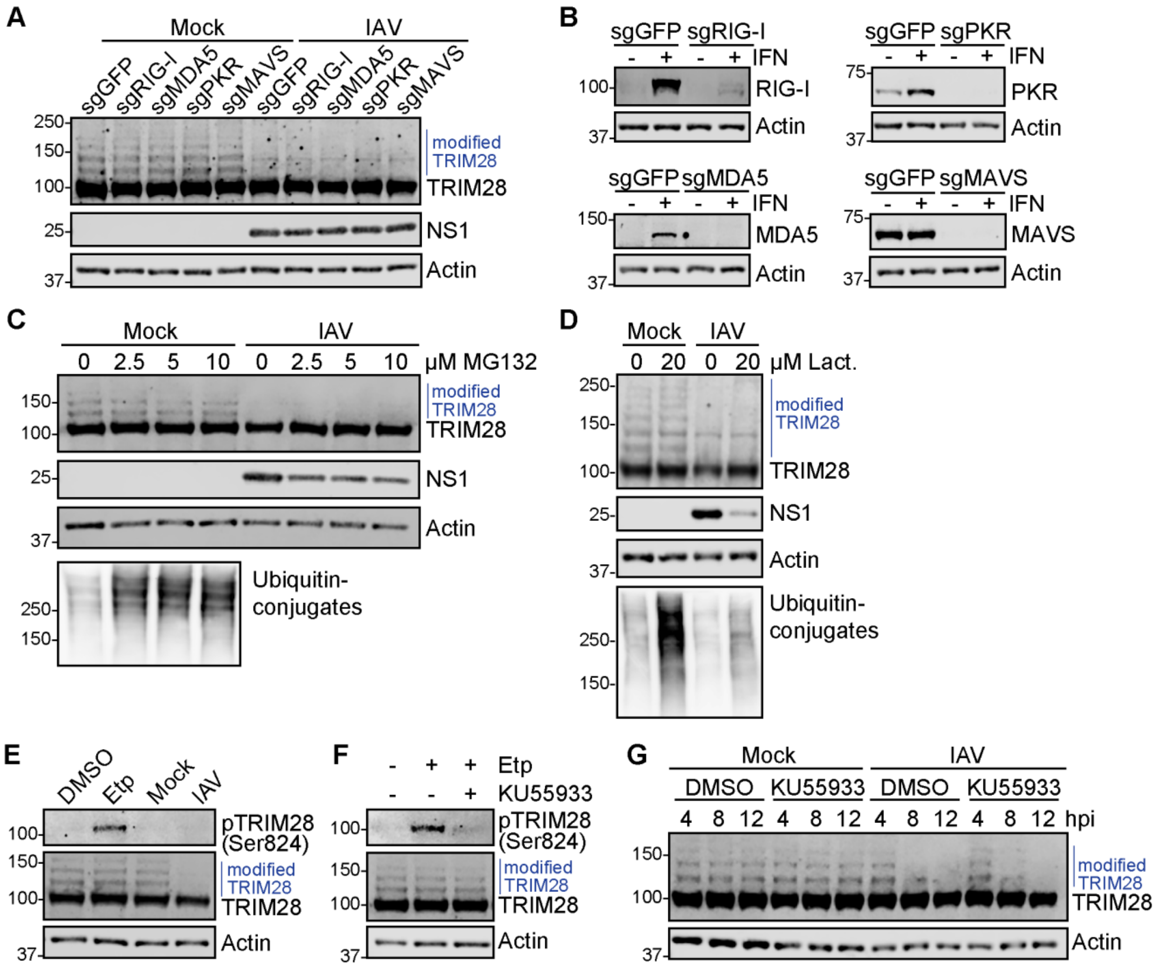

Fig. S1. Loss of SUMOylated TRIM28 During Infection is Independent of Viral RNA Sensing, Degradation by the Proteasome and Canonical DNA Damage Responses. (**A**) A549 cells were transduced with lentiviruses expressing Cas9 and three different sgRNAs targeting RIG-I, MDA5, PKR, MAVS, or GFP (negative control). Cell pools were subsequently infected with IAV (MOI = 5 PFU/cell) for 12h, and cell lysates were subjected to western blot analysis for the indicated proteins. Data are representative of at least two independent experiments. (**B**) Cells generated for (A) were treated with IFNα2 (1000 U/ml, 16h) and processed for western blot to assess knockout efficiency. (**C**-**D**) A549 cells were infected with IAV at MOI = 5 PFU/cell and MG132 (C) or lactacystin (D) was added at the indicated concentrations 1h (MG132) or 2h (lactacystin) later. At 12 hpi, cell lysates were prepared and subjected to western blot analysis for the indicated proteins. Data are representative of at least two independent experiments. (**E**) A549 cells treated with etoposide (Etp, 10 µM, 2h) or infected with IAV (MOI = 5 PFU/cell, 12h), or (**F**) pre-treated with the ATM kinase inhibitor KU55933 (10 µM, 1h) before treatment with Etp (10 µM, 2h) in the presence or absence of KU55933 (10 µM), were lysed and analysed by western blot. Data are representative of at least two independent experiments. (**G**) A549 cells were pre-treated with KU55933 (10 µM, 1h) or DMSO, before infection with IAV (MOI = 5 PFU/cell) in the presence of KU55933 (10 µM) or DMSO. Cells were lysed at the indicated time points and subjected to western blot analysis for TRIM28 and actin. Data are representative of at least two independent experiments.

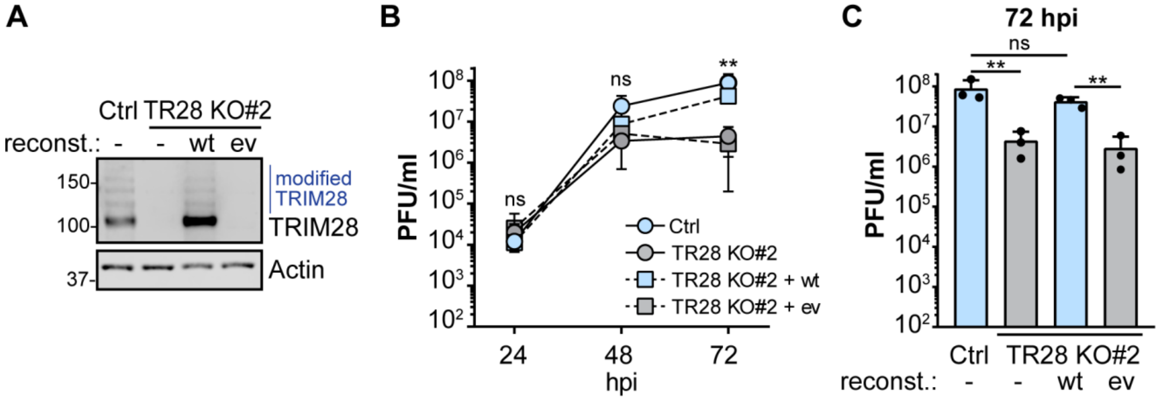

Fig. S2. Additional Validation of TRIM28 Specificity Using an Independent Knock-Out Clone. (**A-C**) The same experiments as described for Figures 3A, C, and D were performed using an independent CRISPR/Cas9-edited TRIM28-KO A549 cell clone (TR28 KO#2) and control. (**A**) Western blot analysis of TRIM28 knock-out A549 cells (TR28 KO#2), or non-targeted control A549 cells (Ctrl), reconstituted with TRIM28 or empty vector (ev) by lentiviral transduction. Data are representative of at least two independent experiments. (**B-C**) Cells described in A were infected with IAV at MOI = 0.001 PFU/cell, and supernatants were collected at the indicated times prior to titration (growth curve plotted in (B); 72 hpi only plotted in (C)). Mean values from three independent experiments are plotted, with error bars representing standard deviations. For panel (C), individual data points are shown in addition. For panels B-C, significance was determined by one-way ANOVA (**$p < 0.01$; ns, non-significant).

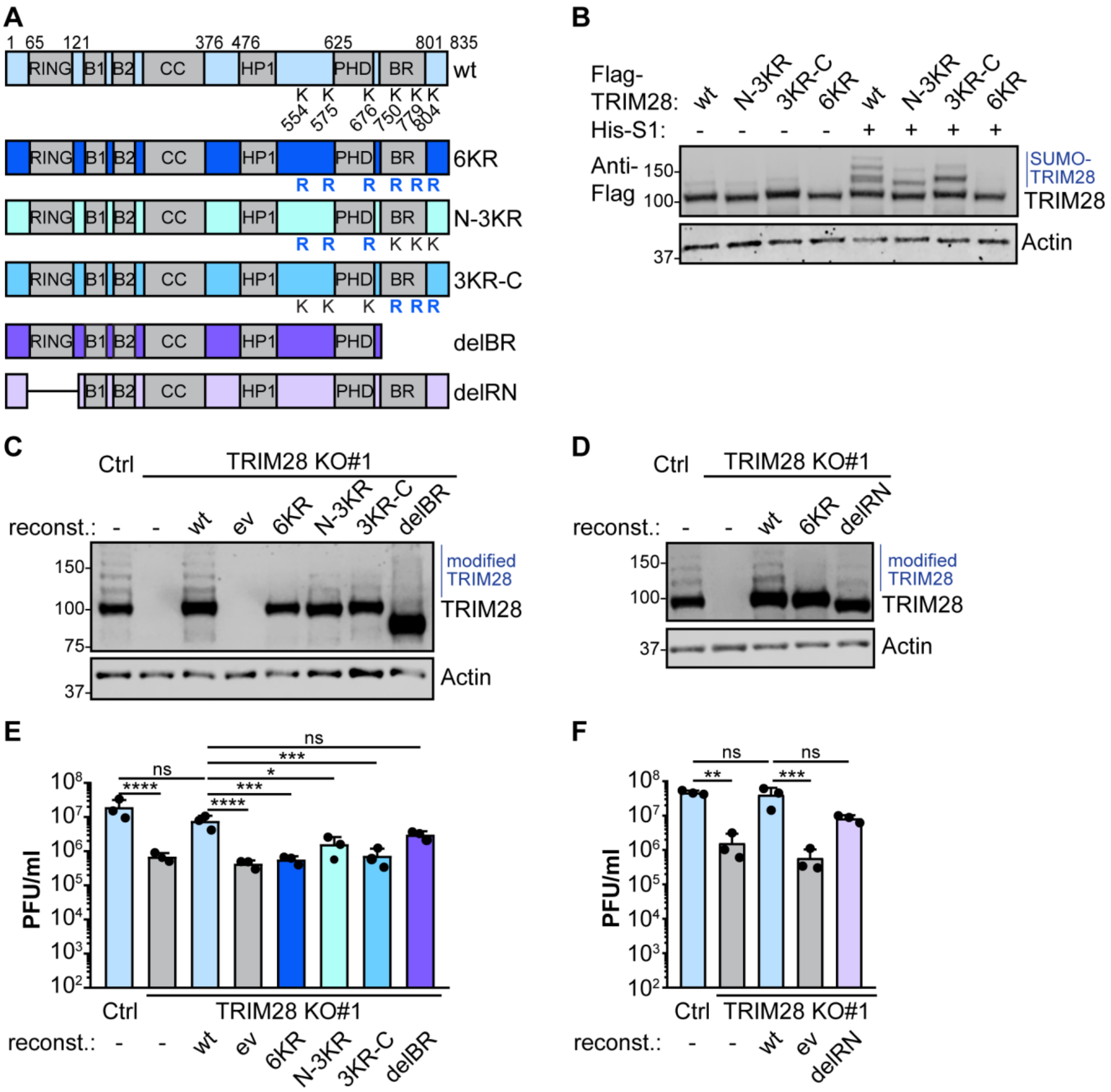

Fig. S3. Functional Analysis of Various TRIM28 Mutants. (**A**) Schematic representation of the indicated TRIM28 mutants (partial SUMOylation-deficient constructs, TRIM28 N-3KR and TRIM28 3KR-C; and deletion mutants lacking the bromo domain (delBR) or the RING domain (delRN). (**B**) Co-transfection of Flag-tagged constructs expressing wt TRIM28, TRIM28 N-3KR, TRIM28 3KR-C, or TRIM28-6KR together with His-SUMO1 (His-S1) into 293T cells. After 24h, cells were lysed and subjected to western blot analysis using the indicated antibodies. Data are representative of at least two independent experiments. (**C-D**) Western blot analyses of control A549 cells (Ctrl) and TRIM28-KO A549 cells (KO#1) reconstituted with different TRIM28 mutants or an empty control vector (ev). Data are representative of at least two independent experiments. (**E-F**) Cells shown in (C-D) were infected with IAV at MOI = 0.001 PFU/cell for 72h prior to titration of supernatants. Each dot represents a single experiment, and bars show mean and SD of three independent experiments**.** Significance was determined by one-way ANOVA (**p* < 0.01; ***p* < 0.01; ****p* < 0.001; *****p* < 0.0001; ns, non-significant). Note: control data in (F) originate from the same experiments as shown in Figure 3H as the experiments were performed in parallel.

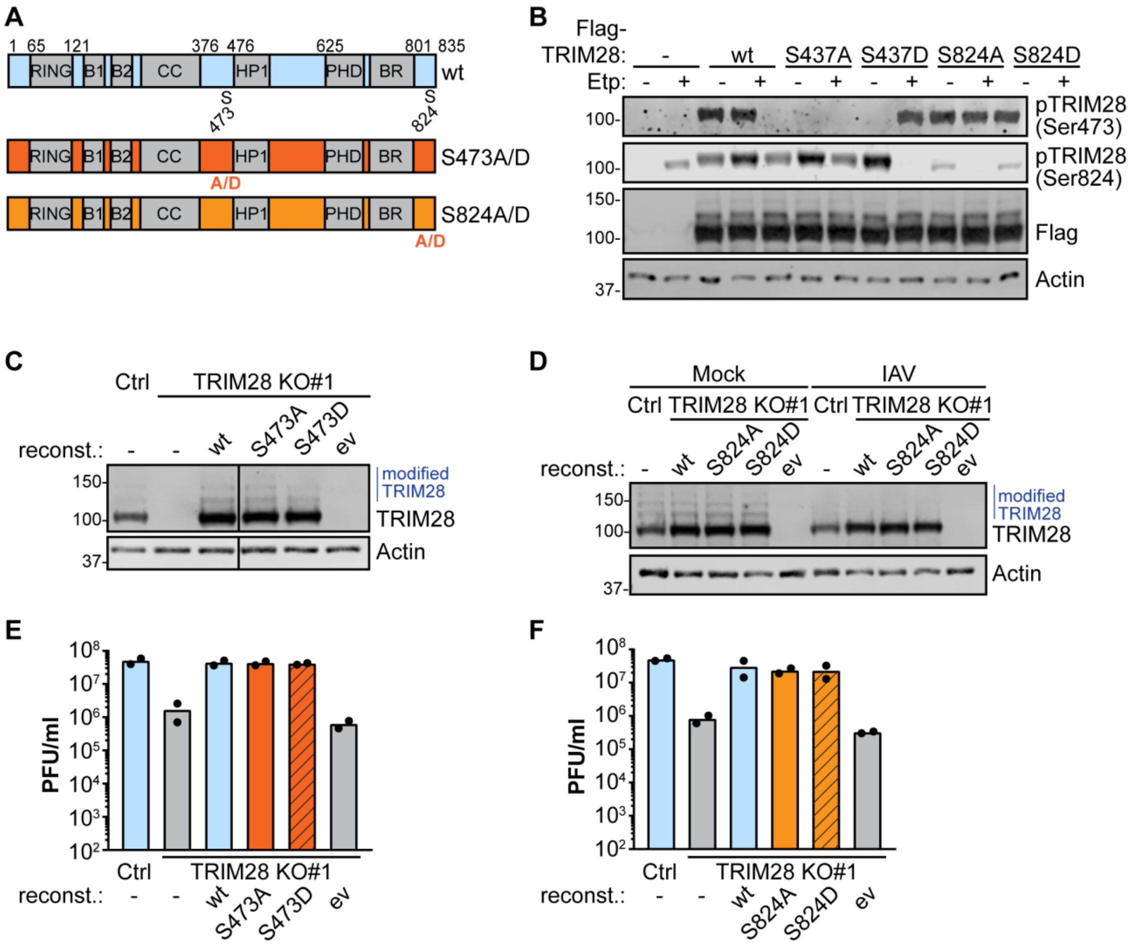

Fig. S4. Functional Analysis of TRIM28 Phosphorylation Mutants. (**A**) Schematic representation of the indicated TRIM28 mutants: phospho-mimetic (D) or phospho-ablation (A) mutants at serines 473 or 824. (**B**) Transfection of Flag-tagged constructs expressing wt TRIM28 or the indicated mutant into 293T cells. After 22h, cells were treated with etoposide (Etp, 10 µM) for 2h prior to being lysed and subjected to western blot analysis using the indicated antibodies. Data are representative of at least two independent experiments. (**C-D**) Western blot analyses of control A549 cells (Ctrl) and TRIM28-KO A549 cells (KO#1) reconstituted with the different TRIM28 mutants or an empty control vector (ev). In (C), all samples were run on the same blot, but irrelevant samples were cropped from the image (indicated by black line). Cells in (D) were additionally infected with IAV at MOI = 5 PFU/cell for 12h. Data are representative of at least two independent experiments. (**E-F**) Cells shown in (C-D) were infected with IAV at MOI = 0.001 PFU/cell for 72h prior to titration of supernatants. Each dot represents a single experiment, and bars show means of two independent experiments.

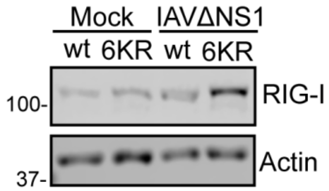

Fig. S5. Cells Expressing SUMOylation-Deficient TRIM28 are Primed for Triggering Enhanced Interferon Responses. TRIM28-KO A549 cells (KO#1) reconstituted with wt TRIM28 or TRIM28-6KR were mock or IAVΔNS1-infected (MOI = 5 PFU/cell, 10h) before being lysed and analyzed by western blot for the indicated proteins. Data are representative of at least two independent experiments.

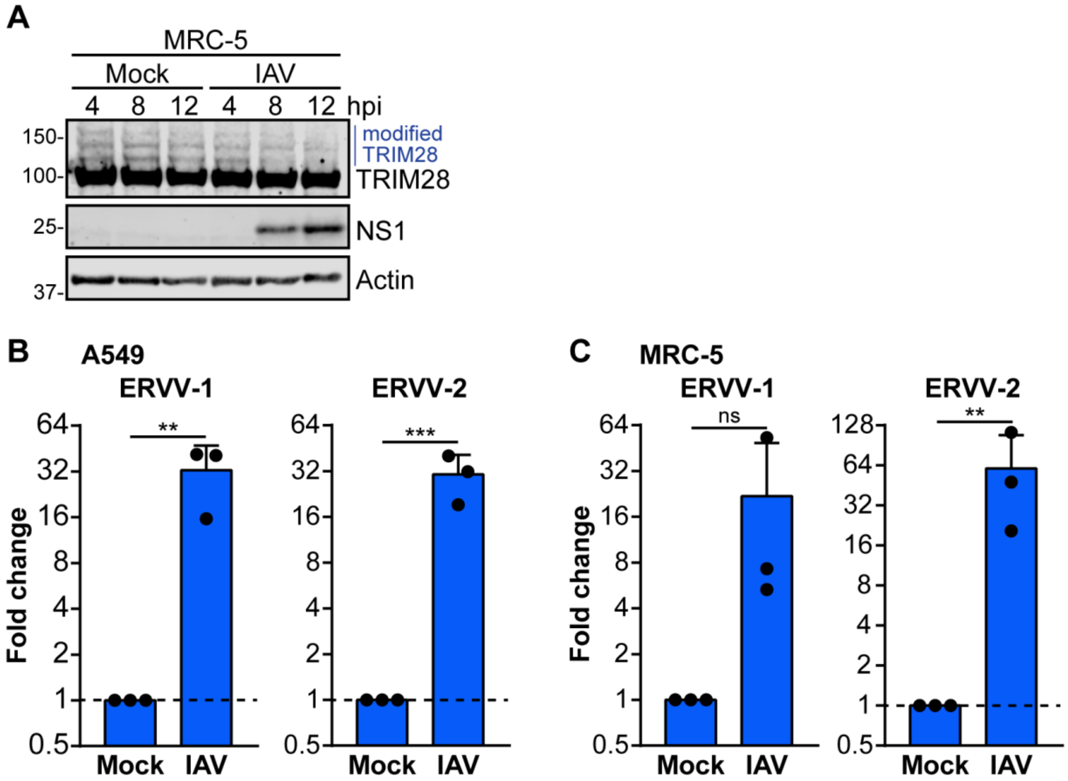

Fig. S6. Infection-Induced Loss of SUMOylated TRIM28 and Upregulation of ERVs in Human Primary-like Cells. (**A**) MRC-5 cells were infected with IAV (MOI = 5 PFU/cell) and harvested at the indicated times post-infection prior to western blot analysis for the indicated proteins. Data are representative of at least two independent experiments. (**B**-**C**) A549 (B) or MRC-5 (C) cells were infected with IAV (MOI = 5 PFU/cell) and harvested at 8h post-infection prior to RT-qPCR analysis for the indicated genes. Bars represent mean values and standard deviations of three independent experiments (each dot represents one replicate). Significance was determined by unpaired t-test (***p* < 0.01; ****p* < 0.001; ns, non-significant). Dashed lines indicate limit of detection.

Additional Dataset S1
Results from SUMO Proteomic Experiments with IAV

Additional Dataset S2
Results from SUMO Proteomic Experiments with IBV

Additional Dataset S3
Results from SUMO Proteomic Experiments with IAVΔNS1

Additional Dataset S4
Overlap Analysis of SUMO Target Proteins Modulated During IAV, IBV, and IAVΔNS1 Infections

Additional Dataset S5
Transcriptome Analysis Comparing TRIM28-6KR vs wt TRIM28 in Uninfected Conditions

Additional Dataset S6
Transcriptome Analysis Comparing TRIM28-6KR vs wt TRIM28 following IAV Infection

Additional Dataset S7
Analysis of Transposable Elements

Additional Dataset S8
Primer Sequences
